# ELDA: real-time functional drug profiling in acute lymphoblastic leukemia

**DOI:** 10.3389/fonc.2026.1685447

**Published:** 2026-03-20

**Authors:** Samara Sousa Mariano, Leandro Henrique de Paula Assis, Juliana Ronchi Correa, Rafael Renatino Canevarolo, Natacha Azussa Migita, Praneeth Reddy Sudalagunta, Amilcar Cardoso de Azevedo, João Meidanis, Silvia Regina Brandalise, Ariosto Siqueira Silva, José Andrés Yunes

**Affiliations:** 1 Leukemia biology laboratory, Boldrini Children’s Center, Campinas, Brazil; 2 Graduate Program in Genetics and Molecular Biology, Institute of Biology, University of Campinas, Campinas, Brazil; 3 Department of Metabolism and Physiology, H. Lee Moffitt Cancer Center and Research Institute, Tampa, FL, United States; 4 Institute of Computing, University of Campinas, Campinas, Brazil; 5 Department of Translational Medicine, State University of Campinas, Campinas, Brazil

**Keywords:** acute lymphoblastic leukemia, *ex vivo* drug screening, PDX models, pediatric leukemia, time-lapse microscopy

## Abstract

**Introduction:**

Pediatric acute lymphoblastic leukemia (ALL) has cure rates exceeding 80%, yet approximately 20% of patients experience relapse, for whom treatment remains challenging. The biological and clinical heterogeneity of ALL complicates therapeutic decision-making, especially in relapse. A functional, patient-specific tool to identify effective drugs could complement current stratification approaches and potentially optimize therapy selection.

**Methods:**

We developed ELDA (*Ex vivo* Leukemia Drug Advisor), a novel drug screening platform based on time-lapse bright-field microscopy. Primary ALL cells were co-cultured with human bone marrow stromal cells in a three-dimensional collagen matrix, replicating a physiologically relevant microenvironment. Candidate drugs were added, and wells were imaged every 30 min for 96 h. A custom image analysis algorithm quantified viable cells by detecting subtle cell membrane movements, enabling real-time monitoring of treatment response.

**Results and discussion:**

ELDA’s results demonstrated strong concordance with established cytotoxicity assays, including MTT and Calcein-AM, validating its performance *in vitro*. Drug profiling of 25 pediatric ALL samples against a panel of 50 clinically relevant and experimental agents revealed distinct sensitivity and resistance patterns for each patient. Notably, heterogeneity was observed even among cases sharing the same genetic alterations, underscoring the limitations of genotype-based therapeutic predictions alone. Drugs with similar mechanisms of action exhibited correlated activity profiles, confirming the biological consistency of the assay. Importantly, ELDA-predicted differences in sensitivity to dexamethasone and vincristine were validated *in vivo*, further supporting its translational relevance. ELDA is a standardized and validated *ex vivo* drug screening methodology capable of generating individualized drug response profiles for pediatric ALL. Beyond its investigational potential in precision oncology, ELDA offers a platform for preclinical evaluation of novel agents and drug combinations.

## 1 Introduction

Acute lymphoblastic leukemia (ALL) is the most common malignancy in children and adolescents, accounting for 25% of pediatric cancers (1, 2). ALL originates from T-cell (T-ALL) or B-cell (B-ALL) precursors, comprising approximately 15% or 85% of cases, respectively. The presence of genetic abnormalities such as aneuploidies, chromosomal translocations, and certain mutations is instrumental for the classification of ALL into several “molecular subtypes,” which may be associated with different clinical responses (3, 4).

Treatment of pediatric ALL is based on chemotherapy with corticosteroids (prednisolone, dexamethasone), antimetabolites (methotrexate, L-asparginase, 6-mercaptopurine, 6-thioguanine, cytarabine), anthracyclines (daunorubicin, doxorubicin), vincristine, and cyclophosphamide (5, 6). Despite the high cure rate for childhood ALL, exceeding 80% in developed countries, relapse, which affects 20% of patients, remains the leading cause of cancer-related death in children and adolescents worldwide (7, 8), and its treatment remains a challenge (9–12).

Intrinsic resistance of leukemic cells to the chemotherapy drugs used in the treatment is the main cause of relapse. This concept is confirmed by the strong association between the delay in the elimination of leukemic cells during the first months of treatment and the occurrence of relapses. It is also confirmed by *in vitro* experiments, in which ALL cells harvested at diagnosis are evaluated for resistance to increasing doses of drugs used in the clinic: dexamethasone, prednisolone, L-asparaginase, and vincristine. Patients whose leukemic cells are more resistant to these drugs are more likely to experience disease relapse (13–17) even when considering only patients from a defined subgroup, such as those at high risk (18) and those with the t(9;22)(BCR/ABL1) (19).

In certain studies, the *in vitro* resistance profile to prednisolone, vincristine, and L-asparaginase (PVA) was an independent prognostic factor for predicting relapses (20–22), although not for late relapses occurring after 2.5 years from diagnosis (21). However, this prognostic strategy was abandoned in favor of minimal residual disease (MRD) assessment, which performs a similar evaluation conducted *in vivo* (23, 24). Nonetheless, *in vitro* assays retain value in identifying potential therapeutic agents for relapsed or refractory ALL. Indeed, *in vitro* drug screening assays have played a fundamental role in the discovery of novel drugs for specific subtypes of ALL. For instance, TCF3-HLF-positive ALL has been shown to be sensitive to venetoclax (25, 26), while pre-B-ALL exhibits sensitivity to dasatinib (27) and T-ALL to dasatinib and temsirolimus (28, 29).

Methods for *in vitro* drug screening in ALL are mostly based on regular liquid cell culture of ALL cells, with or without co-culture on a bone marrow stromal cell (BMSC) layer. Cell viability is measured at pre-determined time points, usually after 72 or 96 h of drug exposure. In monoculture assays, ALL cell viability can be evaluated using the MTT (3-(4,5-dimethylthiazol-2-yl)-2,5-diphenyltetrazolium bromide) reagent, which measures mitochondrial activity and is indicative of cell viability (29). In co-culture assays, viable ALL cells can be labeled with Calcein-AM and differentiated from MSC by image analysis (25) or flow cytometry (30, 31). These are endpoint methods, usually run with 100,000 or 20,000 ALL cells per well in 96- or 384-well plates, respectively.

Here, we present a new method for drug screening in primary ALL cells, based on the co-culture of ALL cells and stromal cells into a collagen matrix. Viability of ALL cells, and discrimination from stromal cells, is obtained by analyzing refringence and membrane movements in time-lapse microscopy. Results obtained by this method, named ELDA for *Ex vivo* Leukemia Drug Advisor, were comparable to those obtained with MTT and Calcein-AM, but it uses only 4,000 ALL cells per well (384-plate), needs no cellular dye, and measures cellular viability in real time.

## 2 Materials and methods

### 2.1 Patient samples

Primary human ALL cells were obtained from cryopreserved bone marrow aspirates of pediatric patients treated at Centro Infantil Boldrini. Informed consent was obtained from the patients and their guardians. The study was approved by the Institutional Research Ethics Committee under CAAE 34601120.7.0000.5376. ALL cells were expanded in NSG-immunodeficient mice, and the patient-derived xenografts (PDX), at passage 1, were cryopreserved in liquid nitrogen. Frozen PDX samples were thawed and used for the experiments. The use of animals for PDX generation and *in vivo* experiments (see below) was approved by the Boldrini Animal Care and Use Committee under protocol number 0011/2020.

### 2.2 TERT-immortalized mesenchymal stem cells

TERT-immortalized mesenchymal stem cells (T-MSCs) were obtained from Dr. Dario Campana (32). T-MSCs were cultured in RPMI-1640 medium (Cultilab, Campinas, Brazil) with 10% fetal bovine serum (FBS). Semi-confluent T-MSC cultures were trypsinized using trypsin/EDTA (Sigma-Aldrich, St. Louis, MO), and cells were washed twice with FBS. T-MSCs were used for the experiment from the third to the tenth passage.

### 2.3 ELDA assay

One and a half million ALL cells (4,000 cells/well) and 40,000 T-MSCs (100 cells/well) in AIM-V culture medium (Invitrogen, Waltham, MA) supplemented with 1.4 mg/mL of type 1 collagen (PureCol^®^ - Advanced Biomatrix, San Diego, CA) were seeded in a 384-well plate (9 µL/well). The plate was spun down and kept 1 h at 37 °C in a CO_2_ incubator for gelification, then 73 µL of AIM-V with 10% FBS was added to each well, and the plate was returned to the incubator overnight. Subsequently, using an EpMotion 5075 pipetting robot (Eppendorf, Hamburg, Germany), drugs were added at five different concentrations with a dilution factor of 1:5, in duplicate (technical replicates). The experimental setup included negative controls with vehicle (DMSO) and positive controls for each drug, represented by the RS4;11 cell line at the highest drug concentration. RS4;11 cells were seeded at 1,300 cells per well, together with 34 T-MSCs per well.

The plate was placed in an EVOS M7000 Cell Imaging Microscope (Thermo Fisher Scientific, Waltham, MA) to capture images of each well every 30 min over a 96-h period. Images were acquired in bright-field and phase-contrast modes using a 5× objective (Olympus, Tokyo, Japan). Each image has a resolution of 2,048 × 1,536. Throughout the imaging process, an attached incubator maintained the cells at a constant temperature of 37°C, with a CO_2_ concentration of 5% and humidity levels exceeding 80%. This controlled environment ensured optimal conditions for cell viability and growth throughout the duration of the experiment.

For digital image analysis, we used the EMMA software created by one of the authors, as described (33–35). In essence, this software distinguishes between living and dead cells by analyzing the membrane movement of ALL cells across consecutive images. EMMA is based on ImageJ and incorporates the TurboReg plugin (36) for alignment of bright-field image stacks acquired at 30-min intervals for the individual wells, correcting translational motion such as plate drift and vibration. The ImageJ background subtraction function (37) was then applied, rendering cells as bright objects against a uniform dark background. The RunningZProjector plugin (gvondassow.com/Research_Site/Methods_files/Running_ZProjector2.java) was used to detect motion and small variations in cell membranes. It detects the maximum pixel intensity across a six-image (3-h) sequence. The analyzed image was then subtracted from the maximum pixel intensity projection, resulting in an image where actively moving membranes appeared as bright rings. ImageJ’s Gaussian blur filter was used to convert these bright rings into spots that cover the entire cell and to produce the overlaid images (red color) shown in this article. Although individual cells were detected, viability was quantified using the sum of their pixel area rather than cell number. Given that the signal is always normalized by the initial time point of the same well, the variation in the initial number of cells between wells, as long as it is not too large (e.g., too many cells in a well would lead to over confluency, etc.), would not affect the readings of the experiment. By applying this algorithm to all images acquired for each well, it becomes feasible to quantify the effects of drug concentration and exposure time in a non-destructive manner, without the need to separate T-MSCs from ALL cells. At the conclusion of the 96-h period, a total of 192 images was captured per well, amounting to 73,728 images per 384-well plate. For more information about the analysis scripts, refer to Khin et al. (33) and Silva et al. (34) The analysis was executed on a system equipped with an Intel Xeon Gold Processor 5118 CPU @ 2.30 GHz, with 256 GB RAM, requiring 13 h per plate (31 drugs).

Cell viability values underwent two normalization steps: 1) The pixel value corresponding to living cells at a given time point for every individual well is divided by the pixel value of the same well at the beginning of the experiment, which is considered 100% viability. The software uses a sequence of six images to assign whether a cell is alive or dead; thus, the first viability value (in pixels) corresponds to time point 6 (3 h after imaging initiation). This first normalization accounts for variations in the number of cells actually photographed in each well (while the same number of cells/well are seeded, final distribution of cells into the well varies quite considerably); 2) the viability at a given time point for each experimental condition is divided by the mean viability of the negative control (vehicle only) replicates at the same time point. The second normalization accounts for drug-independent cell death or cell proliferation.

Primary ALL cells have a very short viability in culture (31, 38, 39). Quality control consisted of three steps: 1) visual inspection of time-lapse videos, 2) analysis of the control viability curve, and 3) analysis of drug-response curves. Experiments in which control ALL cells initiated time-lapse acquisition, largely non-viable (i.e., static), were considered invalid. The viability curve of the control wells may appear acceptable; however, this can result from highly motile stromal cells displacing nearby dead ALL cells, thereby confounding normalized viability estimates. Visual inspection of time-lapse recordings was therefore mandatory for quality control. When control viability was >60% at 60 h, the assay was considered valid. When viability was <50%, the assay was discarded, as it was deemed poorly representative of the initially seeded cell population. When control viability ranged between 50% and 60%, drug-response viability curves were evaluated, and the assay was considered valid provided that an appropriate dose–response pattern was observed (**Supplementary Figure 1**).

### 2.4 MTT assay

A total of 100,000 PDX ALL/well was cultured with RPMI-1640 in 96-well plates. Cell viability was analyzed using the MTT reduction assay (0.5 mg/mL final concentration, 6 h of incubation) after 72 h of treatment (cytarabine, vincristine, and dexamethasone). The formazan dye formed by the viable cells was dissolved by the addition of acid sodium dodecyl sulfate solution (10% SDS, 0.01 mol/L HCl) and overnight incubation at 37°C in the CO_2_ incubator. Absorbance was measured at 570 nm in a Synergy H1 equipment (BioTek, Winooski, VT).

### 2.5 Calcein-AM

To determine cell viability by an orthogonal method, viable cells were discriminated using the CyQUANT Direct Cell Proliferation Assay (Thermo Fisher Scientific) or an in-house labeling with Calcein-AM (1 µg/mL) and propidium iodide (1 µg/mL). Following the completion of an ELDA test, the equipment was halted, but the plate was kept in the plate holder, inside the incubator chamber, to ensure minimal deviation in terms of position and focus with respect to the last ELDA imaging. The CyQUANT reagent was added to the wells, and the plate was incubated for 1 h at 37 °C and 5% CO_2_. Subsequently, wells were photographed using the GFP filter on the EVOS M7000 microscope, using the same settings as the last ELDA imaging. Similar experiments were performed by adding a mixture of propidium iodide and Calcein-AM instead of CyQUANT, in which case the plate was incubated for only 30 min at 37 °C, and imaging was performed using both GFP and RFP filters on the EVOS M7000 microscope. The discrimination between ALL cells and T-MSCs was achieved based on differences in size and shape, and the quantification of green (live) and red (dead) cells was performed using the CellProfiler software (Broad Institute, Boston, MA) (40) with Intel Xeon Gold Processor 5118 CPU @ 2.30 GHz, 2.29 GHz, 256 GB RAM.

### 2.6 Drugs selected for the screening

An FDA-approved anticancer drug library consisting of 166 drugs (kindly provided by the Developmental Therapeutics Program, NCI) was initially screened across eight distinct primary ALL samples. Each drug was tested at six varying concentrations, employing a 10-fold serial dilution starting at 5 µM. The screening assays were conducted as endpoint assessments, with a duration of 72 h. This pilot screening was performed under identical conditions to the ELDA assay (4,000 ALL cells and 100 T-MSCs per well, with collagen and in AIMV supplemented with 10% FBS); however, viable cells were distinguished using Calcein-AM labeling for live cells and propidium iodide staining for dead cells. The CellProfiler software (Broad Institute, Boston, MA) (38) was used to quantify the number of living cells (green fluorescence) and dead cells (red fluorescence) within each well. Drugs showing no activity in any ALL sample at the highest concentration tested were discarded (57 drugs in total). Drugs with activity at ≤0.5 µM in at least one patient sample were pre-selected (49 drugs discarded, 60 drugs pre-selected). Then, the most active 50 drugs were prioritized, covering also various mechanisms of action, as well as drugs of clinical significance in ALL or those involved in promising clinical trials (e.g., imatinib, vorinostat, thioguanine, and ruxolitinib). Detailed information regarding the selected drugs can be found in **Supplementary Table 1**.

### 2.7 *In vivo* drug testing

Ten million PDX ALL cells were injected into NSG mice. Leukemia engraftment was monitored by flow cytometry. When hCD45^+^ cells over total CD45^+^ (mCD45^+^hCD45) cells reached a median average of 0.5%, mice were randomized into the control, dexamethasone (5 mg/kg, intraperitoneal, from Monday to Friday), and vincristine (0.15 mg/kg, intraperitoneal, every Monday) groups. A total of *n* = 7 mice per treatment group were included in each experiment. Treatment lasted 4 weeks. The percentage of hCD45 in the peripheral blood was monitored weekly. CD45 values between two time points were interpolated linearly based on the straight line connecting the measurements. For survival analysis, a death event was defined as the mouse reaching 25% hCD45 in peripheral blood or exhibiting leukemia-related morbidity (weight loss, lethargy, ruffled fur), whichever occurred first.

### 2.8 Data analysis

Statistical analysis was done with GraphPad Prism V6.0 (GraphPad Software, La Jolla, CA) using Student’s *t*-test, two-way ANOVA with Dunnett’s multiple comparison test, or Pearson’s correlation analysis. *p*-values <0.05 were considered statistically significant and marked as *p < 0.05, **p < 0.01, ***p < 0.001, and ****p < 0.0001. The area under the curve (AUC) and the lethal dose to kill 50% of the cells (LD_50_) were provided by the EMMA software (35).

## 3 Results

**Figure 1:**
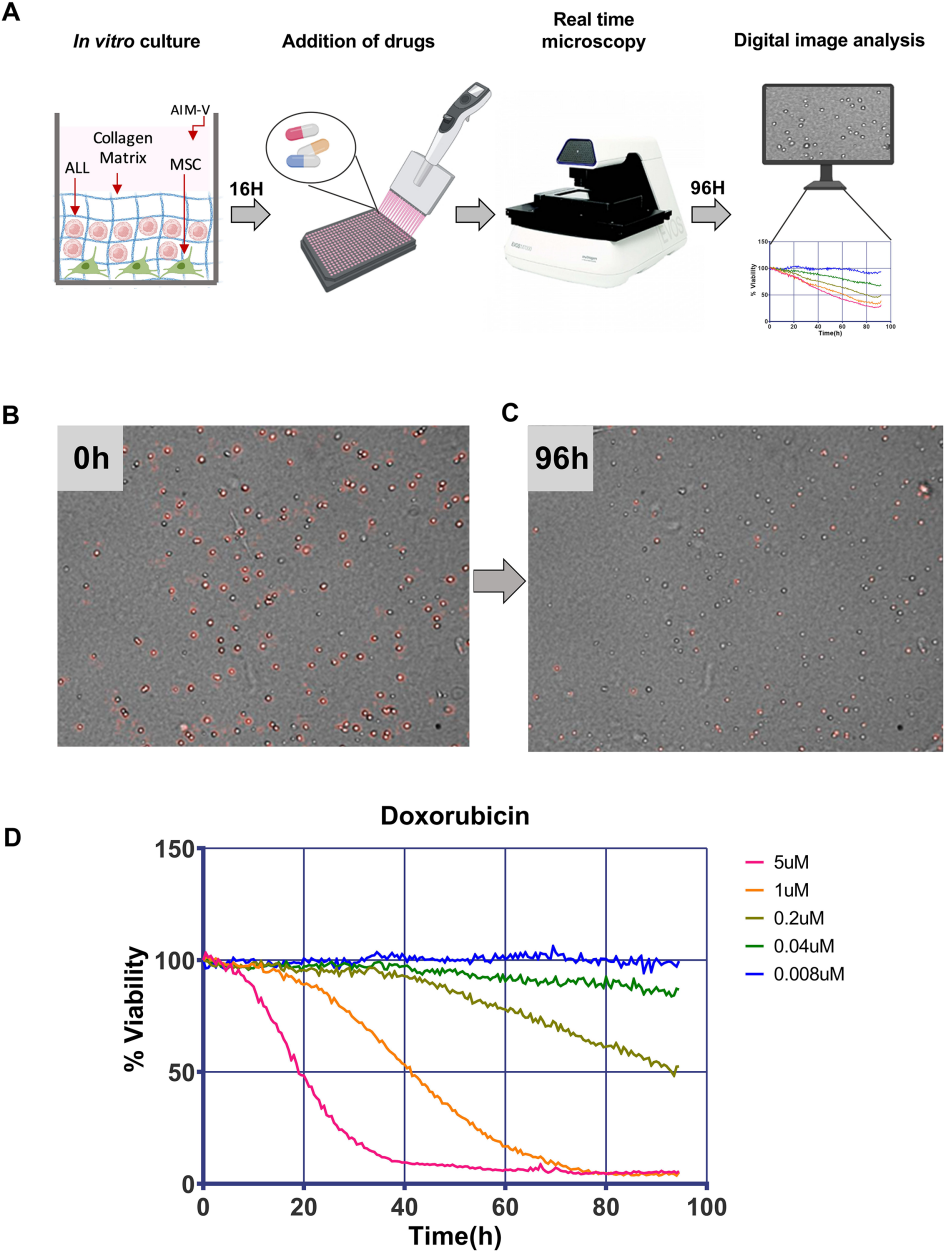
Overview of the *Ex vivo* Leukemia Drug Advisor (ELDA) assay. **(A)** Schematic representation of the four steps of the ELDA assay (Created with BioRender.com). The initial step involves co-culturing of primary ALL cells with a stromal cell line (T-MSC) in a collagen matrix supplemented with culture medium containing FBS. After overnight incubation, drugs at five different concentrations are added to the plate in duplicate. The plate is placed in the onstage incubator of the EVOS system, and each well is photographed every 30 min in bright field, for 4 days. Subsequently, the images are analyzed by a proprietary software that evaluates temporal variations in image sequences in order to provide information about cells, whose positions are kept, to a certain extent, constant by the collagen matrix. Even so, living cells move and exhibit a kind of small membrane movements that are not affected by the collagen matrix, but all movements cease upon cell death. The software discriminates these movements by analyzing spatio-temporal pixel changes, then calculates the sum of the area of all cells showing membrane movements. Since there is considerable variation in the number of cells actually photographed in each well, a normalization is done by dividing each value by the value at time point 6 (3 h after the beginning of the incubation in the EVOS system), which is considered 100% viability. To evaluate the effect of drugs, the percentage of live cells at a given time point for each experimental condition is divided by the mean viability of the 10 control (vehicle only) replicates at the same time point. **(B)** Illustration of digital image analysis using 0.2 µM of daunorubicin. The analysis software pseudocolors live ALL cells in red based on membrane movements. In this example, the majority of cells are alive at time point 0, considered as 100% for normalization. However, after 96 h, nearly all cells are dead **(C)**. **(D)** Example of a dose–response graph using daunorubicin. The graph shows the relative viability of ALL cells in five different concentrations of daunorubicin over the 96-h period. As mentioned above, note that the viability shown is relative to the viability of the same cells, in the same plate, treated with vehicle.

The ELDA assay is an adaptation of the EMMA method developed for multiple myeloma (33–35) and comprises four key execution steps (**Figure 1A**). Firstly, a co-culture is established in a black 384-well plate, combining primary ALL cells, T-MSCs, a type 1 collagen matrix, and AIM-V culture medium supplemented with 10% FBS. The second step occurs 16 h later, with the addition of drugs at five different concentrations. Each 384-well plate accommodates 31 drugs in duplicate, alongside negative control wells comprising vehicle (DMSO) and positive control wells where a cell line is treated with the maximal drug concentration in order to validate drug stability and assay performance.

Following the addition of drugs, the plate undergoes time-lapse microscopy, wherein each well is photographed in phase contrast every 30 min for a duration of 80 to 96 h, using the EVOS M7000 microscope (Thermo Fisher Scientific). At the conclusion of this 96-h period, images undergo analysis using a software developed by Silva et al. (2017) (35), which discerns between living and dead cells based on dynamic changes in cell membrane movement over sequential images. The software is capable of distinguishing leukemia cells from stromal cells based on an optical artifact formed by non-adherent cells in phase contrast: a lens-like structure (similar to the appearance used to distinguish live from dead cells in a hemacytometer), in which the cytoplasm appears bright, and the membrane is outlined as a dark ring. In the initial image captured at time zero, ALL cells are identified and pseudo-colored in red (**Figure 1B**). As time progresses, living cells exhibit membrane movements, which alter the quantification of pixels at their specific XY coordinates in sequential images; these locations are consistently colored red by the software. Conversely, cells undergoing death show no membrane movement, resulting in an unchanged spatial pixel count relative to previous images, and are therefore rendered as non-red (**Figure 1C**). Representative videos showing ALL cells that remain viable (**Supplementary Video 1**) or that undergo progressive loss of viability over the course of the experiment (**Supplementary Video 2**) are provided as supplementary material. Applying this algorithm to all consecutive images from each well of the plate, the software can ascertain the added viability of ALL cells over time in a non-destructive manner, eliminating the necessity to add viability reagents or separate stromal from ALL cells. Consequently, dose–response graphs can be generated based on concentration and exposure time, facilitating the calculation of LD_50_ and AUC values for each drug tested (**Figure 1D**).

### 3.1 ELDA standardization

**Figure 2:**
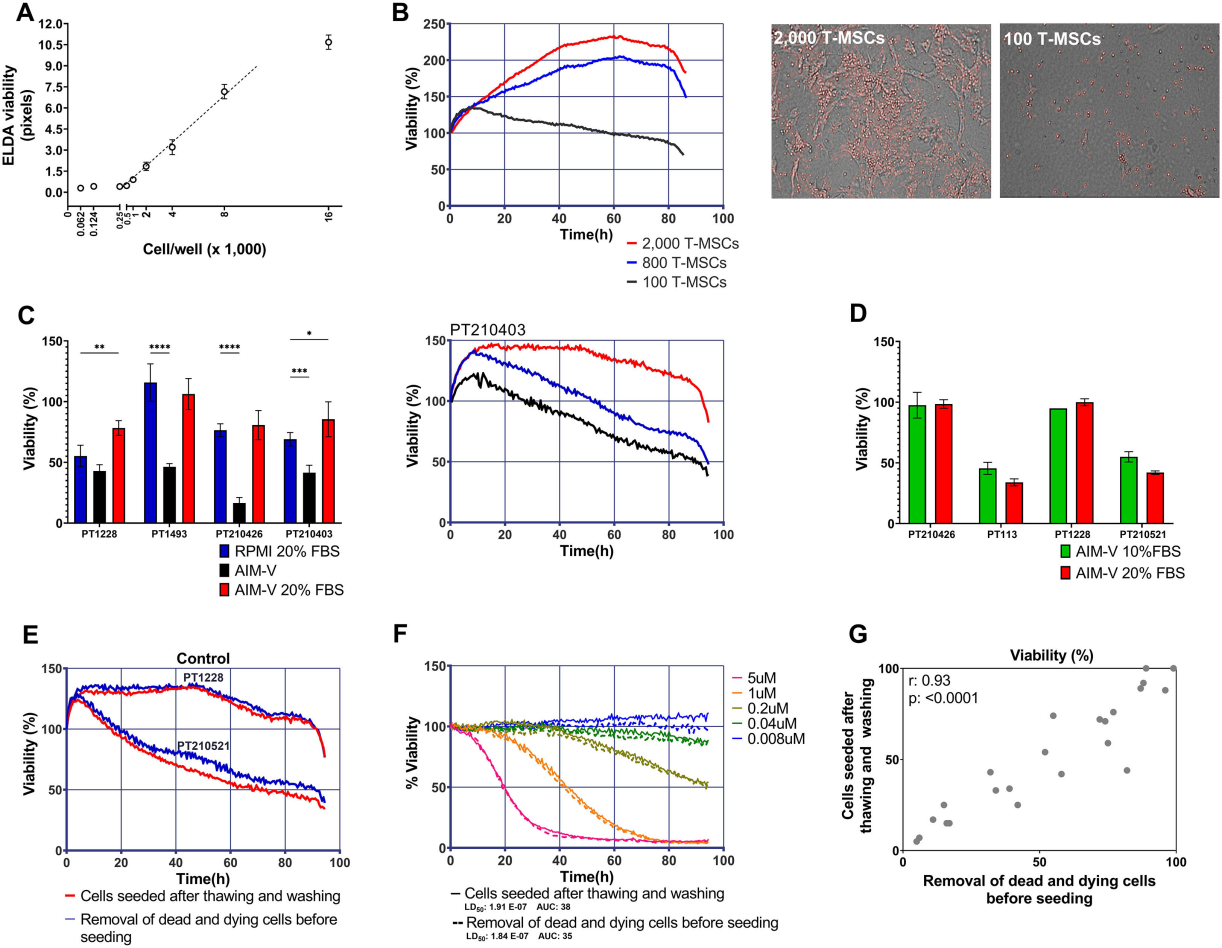
ELDA standardization. **(A)** Determining the number of ALL cells to be seeded in each well. The graph illustrates ELDA’s capability to discern clear differences between 1,000 and 16,000 ALL cells per well. Densities from 62 to 500 cells/well could not be discriminated. **(B)** Standardization of the number of stromal cells per well. Representative graphs and images of ALL cells seeded with 2,000, 800, and 100 MSCs. High numbers of MSCs (2,000 and 800), which are highly motile, can move near ALL cells (whether alive or dead), leading to artifactual increases in apparent cell viability over time. When only 100 MSCs per well were used, minimal interference with ALL cell movement and recorded cell viability was observed. **(C)** Test of different culture media. Cell viability at 96 h of four different pediatric ALL-PDX samples in co-culture with 100 MSCs per well using RPMI-1640 (RPMI) or AIM-V culture media supplemented or not with FBS. The right panel shows cell viability over time for a representative sample. **(D)** Viability of ALL cells after 96 h of culture in AIM-V with 20% FBS vs. AIM-V with 10% FBS. No major differences were observed, regardless of whether the samples maintained good or poor viability over time. Statistics performed by two-way ANOVA with Dunnett’s multiple comparison test (*p* < 0.05). **(E)** Removal of apoptotic and dead cells before ELDA seeding is not necessary. ELDA results for two different patient samples (PT1228 and PT210521) that were subjected or not to the magnetic removal of cell debris, dead cells, and dying cells before seeding. **(F)** Doxorubicin dose–response curves for patient sample PT1228, with or without dead cell removal before seeding. **(G)** Correlation between viability at 96 h for four different ALL samples, according to dead cell removal or not before seeding. Data from a dose–response experiment for doxorubicin. Pearson’s correlation. PT, patient.

To determine the minimal number of ALL cells measurable by the image analysis system, while addressing its reproducibility, we cultured primary ALL at different cell densities, ranging from 62 to 16,000 cells per well, in 10 technical replicates. After collagen gelification, the plate was placed in the onstage incubator of the EVOS system and imaged every 30 min for 4 h. Cell numbers were measured using the EMMA software. As shown in **Figure 2A**, the system could not reliably distinguish between 1,000 from 500 or 62 cells. A geometric progression was apparent between 1,000 and 8,000. At the highest cell density tested (16,000), however, the trend seemed to diminish its slope. Reproducibility remained consistent across replicates.

It is well-established that co-culturing of ALL onto a layer of MSCs prolongs the survival of ALL *in vitro* (38, 41). However, in ELDA, the addition of too many MSCs is not feasible, as the movement of MSCs can also move surrounding ALL cells in the collagen matrix, which may or may not be living ALL cells, flagging dead cells as live cells. When testing the co-culture of ALL with 2,000 MSCs, excessive movement of the stromal cells caused significant overestimation of ALL cell viability. A similar issue occurred using 800 MSCs per well. Therefore, we systematically reduced the number of MSCs until we identified the optimal number of 100 MSCs per well (**Figure 2B**), which preserved stromal support while minimizing motion-related artifacts.

The culture medium plays a crucial role in providing essential nutrients and energy sources and maintaining optimal pH and osmolarity levels for cells *in vitro*. Traditionally, ALL cell lines are cultured using RPMI supplemented with FBS. Primary ALL cells are co-cultured onto bone marrow stromal cells using the serum-free AIM-V medium (25, 42, 43). AIM-V is a nutrient-rich medium formulation based on DMEM and Ham’s F12, enriched with human serum albumin, insulin, transferrin, and a blend of purified non-cytokine factors (44).

The viability of ALL cells cultured in serum-free AIM-V was lower than in RPMI supplemented with 20% FBS. However, when 20% FBS was added to AIM-V, the viability of ALL cells from two out of four patients (50%) was higher than that with RPMI-20% FBS (**Figure 2C**). Subsequent experiments revealed that comparable results could be achieved with AIM-V supplemented with only 10% FBS (**Figure 2D**, **Supplementary Figure 2**).

After performing some ELDA tests, it became evident that ALL cells, with very rare exceptions, did not proliferate *in vitro*, and some lost viability very rapidly, in less than 48 h. Since freezing and thawing may result in dead cells, we tested whether the removal of apoptotic and dead cells using magnetic beads, before cell seeding, would have an impact on ELDA results. However, no differences were observed by removing apoptotic/dead cells, either under drug-free conditions (**Figure 2E**, **Supplementary Figure 3**) or with respect to the LD_50_ and AUC values for doxorubicin treatment (**Figures 2F, G**). These results indicate that dead cells do not interfere with ELDA results. As cellular viability is normalized to the number of living cells (in pixels) at the beginning of time-lapse microscopy, i.e., more than 14 h after cell seeding, those cells that were prone to die had already died, thus making no difference in the experiment, provided that enough number of living cells were available to record the effects of the drugs.

### 3.2 Validation of ELDA results by orthogonal methodologies

The ELDA assay categorizes a cell as viable when it exhibits membrane movements, whereas cessation of movement results in the classification as dead. To validate that movements are indeed specific to living cells and the lack of movements for the dead ones, we used fluorescent dyes to label live and dead cells.

**Figure 3:**
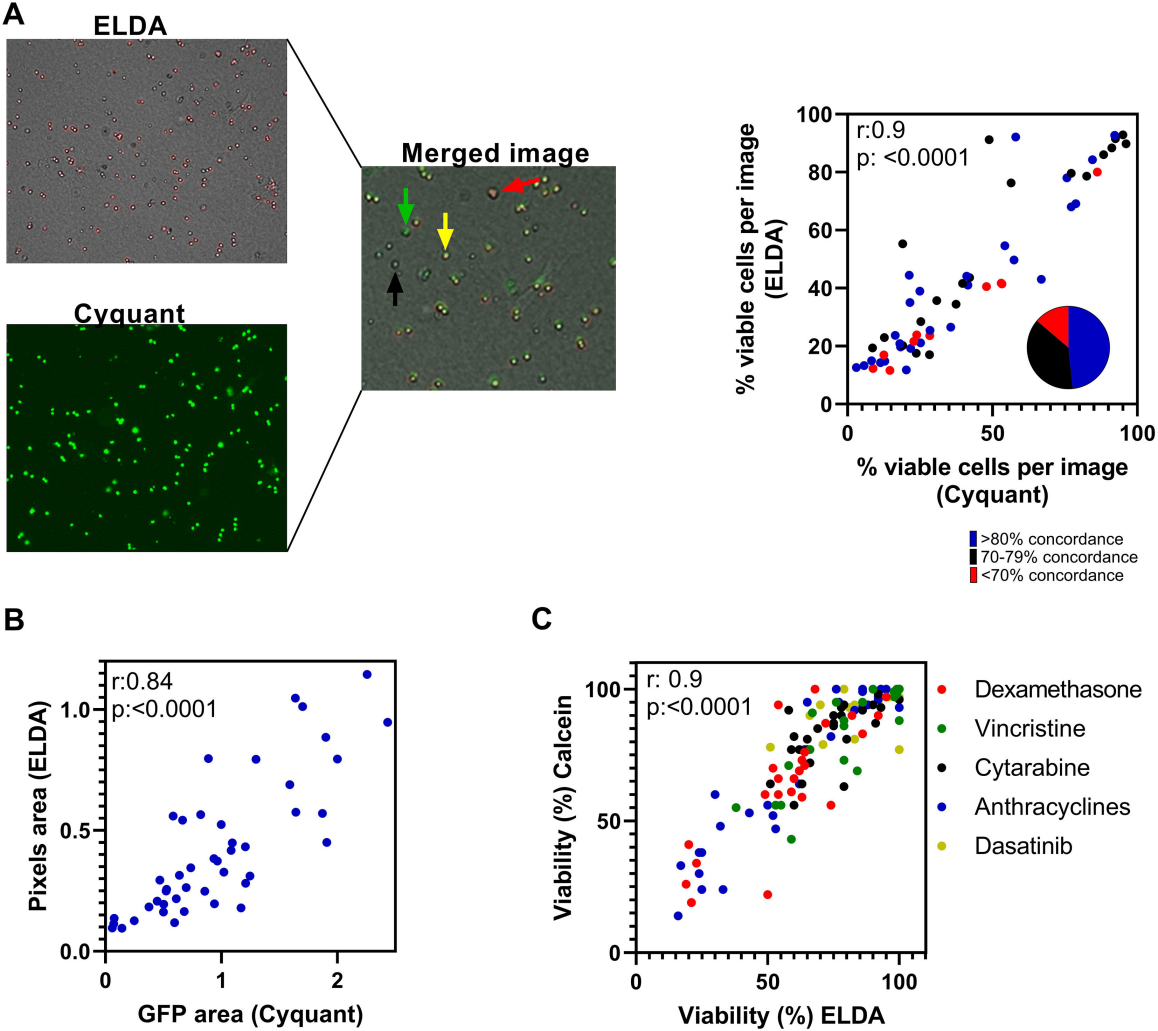
Validation of the ELDA assay by fluorescent detection of viable and dead cells. **(A)** Comparison between the viability of the RS4;11 cells against 31 drugs using ELDA vs. the CyQUANT Direct Cell Proliferation Assay (left panels). The right panel displays merged images from ELDA and CyQUANT. ELDA pseudo-colors live cells in red, while CyQUANT stains live cells in green. Cells appearing yellow or orange correspond to those classified as live by both methods. The correlation graph depicts the percentage of live cells per image according to ELDA and CyQUANT. Each dot corresponds to one of the 62 wells of the 384-well plate, thus to one of the technical replicates corresponding to one of the 31 drugs. The color of the dots indicates the percentage of cells per image, in which both methods agreed whether the cell was alive or dead. The pie chart shows the proportion of agreement between methods for all 62 images analyzed. **(B)** Correlation between the average green fluorescent area (CyQUANT) and ELDA live cell area in pixels. **(C)** Correlation between ELDA cell viability results and viability results obtained by using fluorescence probes (Calcein-AM and propidium iodide) on primary pediatric ALL samples treated with five different drugs, at five different concentrations, over a 72-h period. Anthracyclines: doxorubicin and daunorubicin. Statistical analysis corresponds to Pearson’s correlation.

First, the RS4;11 cell line was cultured in the ELDA system with 31 drugs, in duplicate wells, for a duration of 80 h. Upon completion of the ELDA, the CyQUANT Direct Cell Proliferation Assay kit was used to label viable cells, and the plate was reimaged using a GFP filter. The final phase-contrast image obtained from the ELDA analysis was then merged with the GFP-filtered (green fluorescent) image captured after CyQUANT. While ELDA pseudo-colored live cells in red, CyQUANT labeled live cells in green. Consequently, when both methods agreed that the cells were alive, overlapping regions of red and green resulted in shades of yellow. Conversely, when both methods confirmed cell death, no color was observed. Regions colored red only denoted instances where only ELDA classified cells as viable, while green indicated cells solely identified as viable by CyQUANT. The 62 images generated from the analysis of the 31 drugs, conducted in duplicates, were individually assessed. The level of agreement between ELDA and CyQUANT, i.e., the number of discolored plus yellow cells over the total number of cells, was 78% on average. In nearly 90% of the analyzed images, the level of agreement between ELDA and CyQUANT exceeded or was equal to 70%. Only 13% of the images exhibited an agreement of less than 70% regarding the identification of live and dead cells. Moreover, a robust correlation (*r* = 0.9; *p* < 0.0001) was observed when comparing the cell viability data obtained concurrently through ELDA and CyQUANT analysis across the different images (**Figure 3A**). Additionally, a strong correlation (*r* = 0.84; *p* < 0.0001) was evident between the total area of green fluorescent cells (in pixels) following CyQUANT labeling and the total area of live cells (in pixels) identified by ELDA (**Figure 3B**).

In a similar experimental setup, primary ALL cells were subjected to the analysis. Five primary ALL samples, from different genetic subtypes, were seeded into the ELDA system and exposed to six different drugs—cytarabine, vincristine, dexamethasone, idarubicin, doxorubicin, and daunorubicin—at varying concentrations. Following a 72-h incubation period, ELDA imaging was concluded, and the live and dead cells were distinguished by the addition of Calcein-AM and propidium iodide to the plates. Subsequently, the plates were photographed using appropriate fluorescence filters, and cell viability was assessed using the CellProfiler software, which differentiated between ALL and T-MSC cells based on their size disparity. Consistent with previous findings, a robust correlation (*r* = 0.9; *p* < 0.0001) was observed between cellular viability outcomes obtained via ELDA and those derived from the Calcein-AM labeling method (**Figure 3C**).

**Figure 4:**
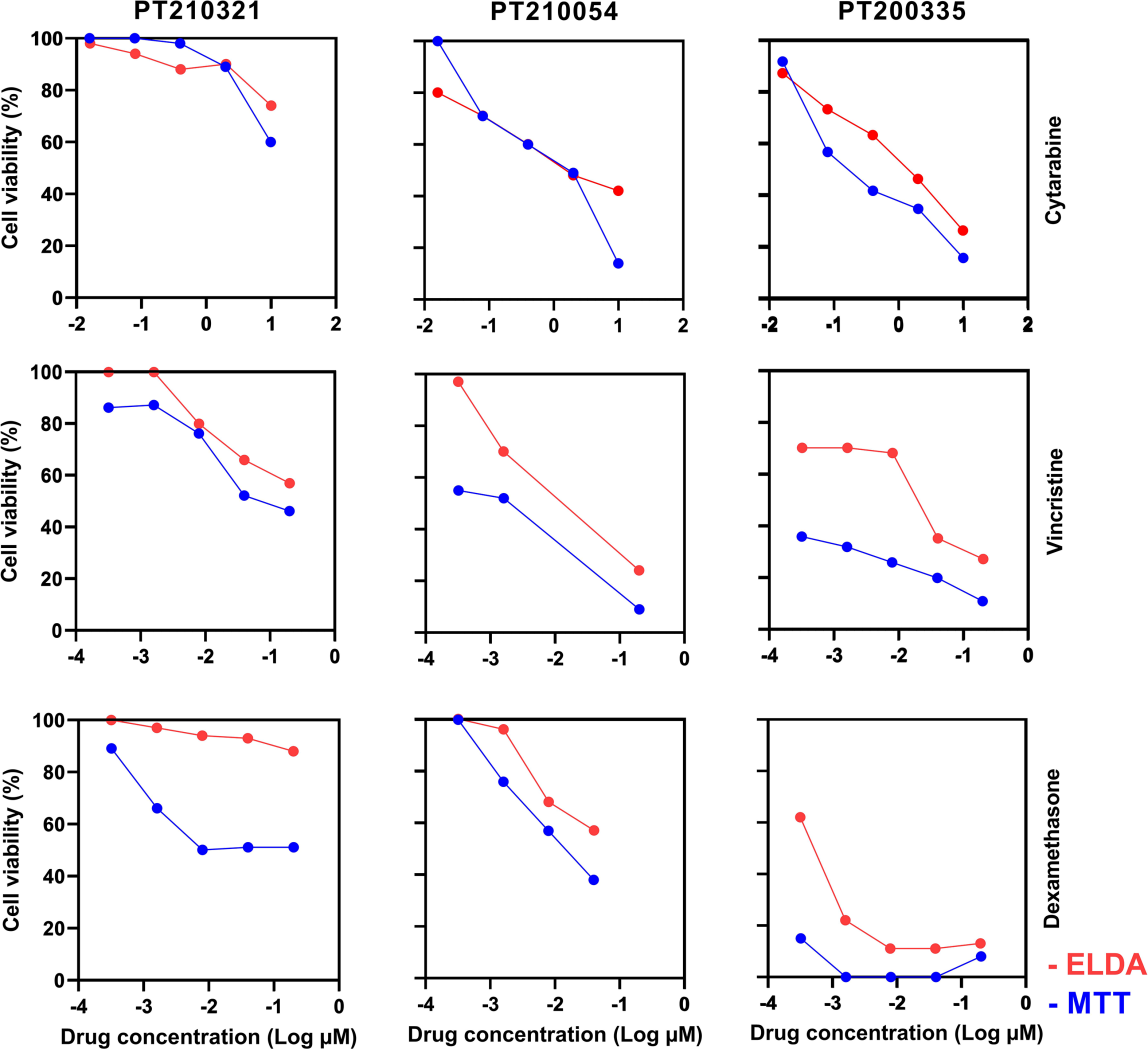
Comparison of drug profiling results obtained with the MTT and ELDA techniques. Samples from three different pediatric ALL PDX (PT210321, PT210054, and PT200335) were evaluated against cytarabine, vincristine, and dexamethasone at five different concentrations. The MTT assay was performed in 96-well plates, using 100,000 cells/well in RPMI-1640 with 10% FBS, as previously described (45). ELDA was done using 4,000 cells/well in 384-well plates. Results collected at 72 h are shown.

Lastly, we conducted a comparative analysis between ELDA and the MTT assay across three primary ALL samples, evaluating five different concentrations of cytarabine, vincristine, and dexamethasone. While a positive correlation between ELDA and MTT viability results was observed, the ALL cells exhibited slightly greater resistance to the tested drugs in the ELDA as compared to MTT (**Figure 4**). To investigate whether the collagen matrix utilized in ELDA could contribute to increased drug resistance in ALL cells, we performed an MTT viability assay with and without collagen. Viability remained highly similar in the presence and absence of collagen (**Supplementary Figure 4**), dismissing any apparent interference of collagen in the differences observed between the ELDA and MTT assays. In line with existing literature reports (25, 46), it is possible that the inclusion of T-MSCs in ELDA, absent in the MTT assay, confers a protective effect on ALL cells. Other possible explanations for this difference are the higher number of ALL cells per well in the MTT assay and the fact that ELDA measures cell movements while MTT measures mitochondrial activity as a means of assessing the numbers of living cells. In conclusion, even though ELDA has been validated for the discrimination of live and dead cells, the results obtained may not perfectly translate to other drug profiling techniques.

### 3.3 Drug screening in 25 pediatric ALL patient samples

To further evaluate the potential of ELDA, we analyzed PDX-derived samples from 31 pediatric ALL cases. The biological and clinical features of these ALL cases are detailed in **Supplementary Table 2**. As mentioned previously, six samples (19%) failed to meet predefined viability quality criteria (negative control viability ≥ 50%) or were already mostly dead at the start of time-lapse recording, and were therefore excluded from further analysis. The remaining 25 samples were uniformly screened against 50 preclinical and clinical compounds (**Supplementary Table 3**). For each drug, we utilized five doses optimized from an initial six-point screen (**Supplementary Table 1**). Notably, the majority of the compounds tested did not exhibit cytotoxic effects on T-MSCs at concentrations lethal to ALL cells (**Supplementary Video 3**), indicating selective drug activity. Bortezomib, carfilzomib, fedratinib, ixabepilone, omacetaxine, panobinostat, and vincristine elicited cytotoxic effects on T-MSCs at the highest concentration (**Supplementary Table 3**, **Supplementary Video 4**).

**Figure 5:**
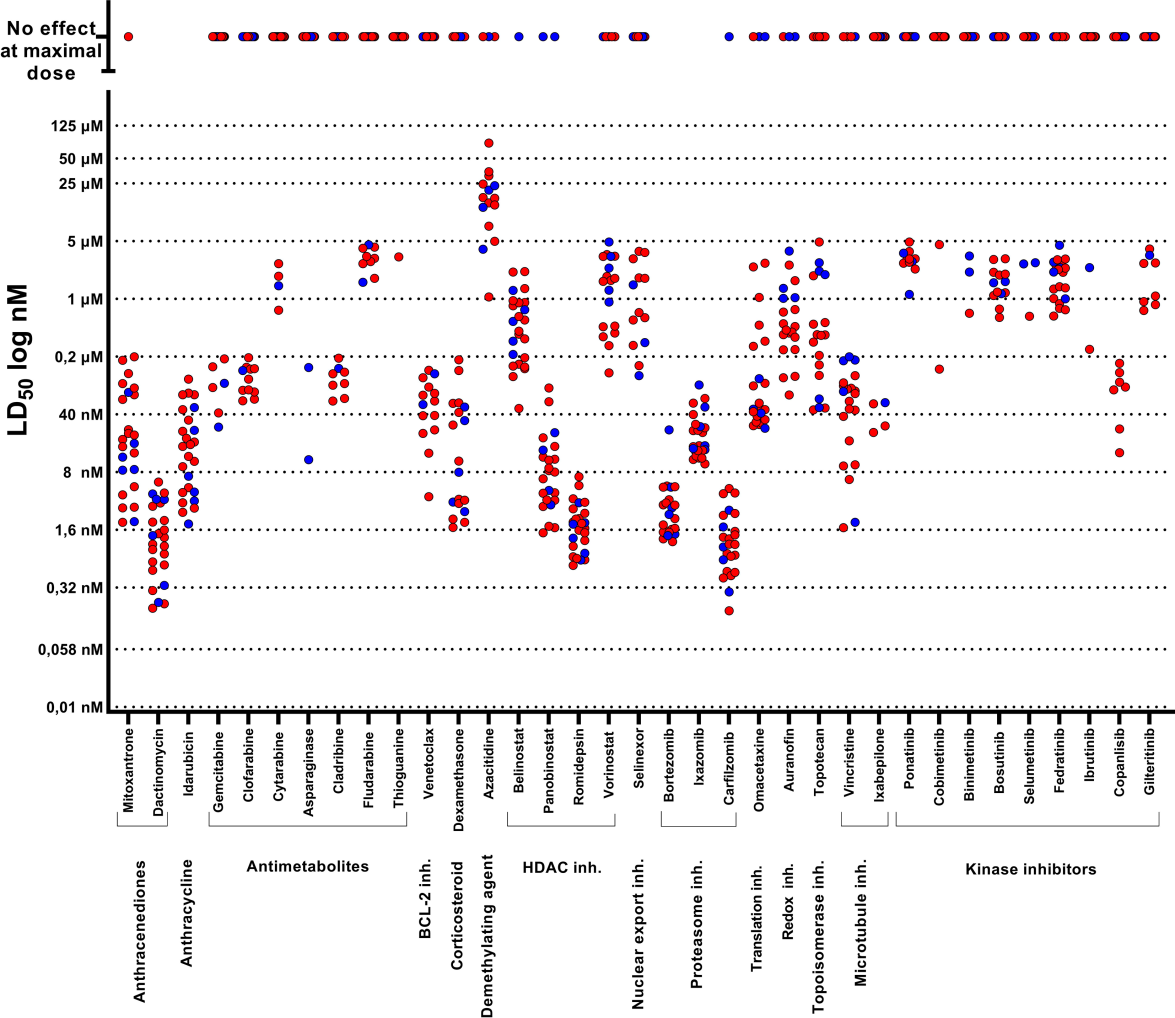
Drug response profiles of 25 pediatric ALL samples according to LD_50_. First-passage patient-derived xenograft (PDX) samples from 25 different ALL cases were screened against 50 compounds at five different concentrations. Each circle corresponds to the LD_50_ value for one of the 25 ALL cases. B-ALL samples were colored red, and T-ALL samples were colored blue. Fifteen drugs were excluded from the graph, as they had no effect at the highest tested dose (cisplatin, glasdegib, niraparib, nelarabine, imatinib, dasatinib, acalabrutinib, idelalisib, zanubrutinib, ruxolotinib, alpelisib, duvelisib, axitinib, capmetinib, and erlotinib).

Compounds such as bortezomib, carfilzomib, and romidepsin demonstrated efficacy at low LD_50_ values (in nM) and exhibited a narrow LD_50_ range in most ALL cases. Conversely, certain conventional cytotoxic agents, such as dexamethasone, mitoxantrone, vincristine, idarubicin, and dactinomycin, as well as panobinostat and omacetaxine, displayed responses distributed over a wider concentration range, with pronounced activity observed in the nanomolar range. On the other hand, most tyrosine kinase inhibitors (TKIs), at the concentrations used (5 µM maximal dose), demonstrated limited efficacy across most ALL cases, and when any efficacy was observed, the LD_50_ values were typically in the micromolar (µM) range (**Figure 5**).

**Figure 6:**
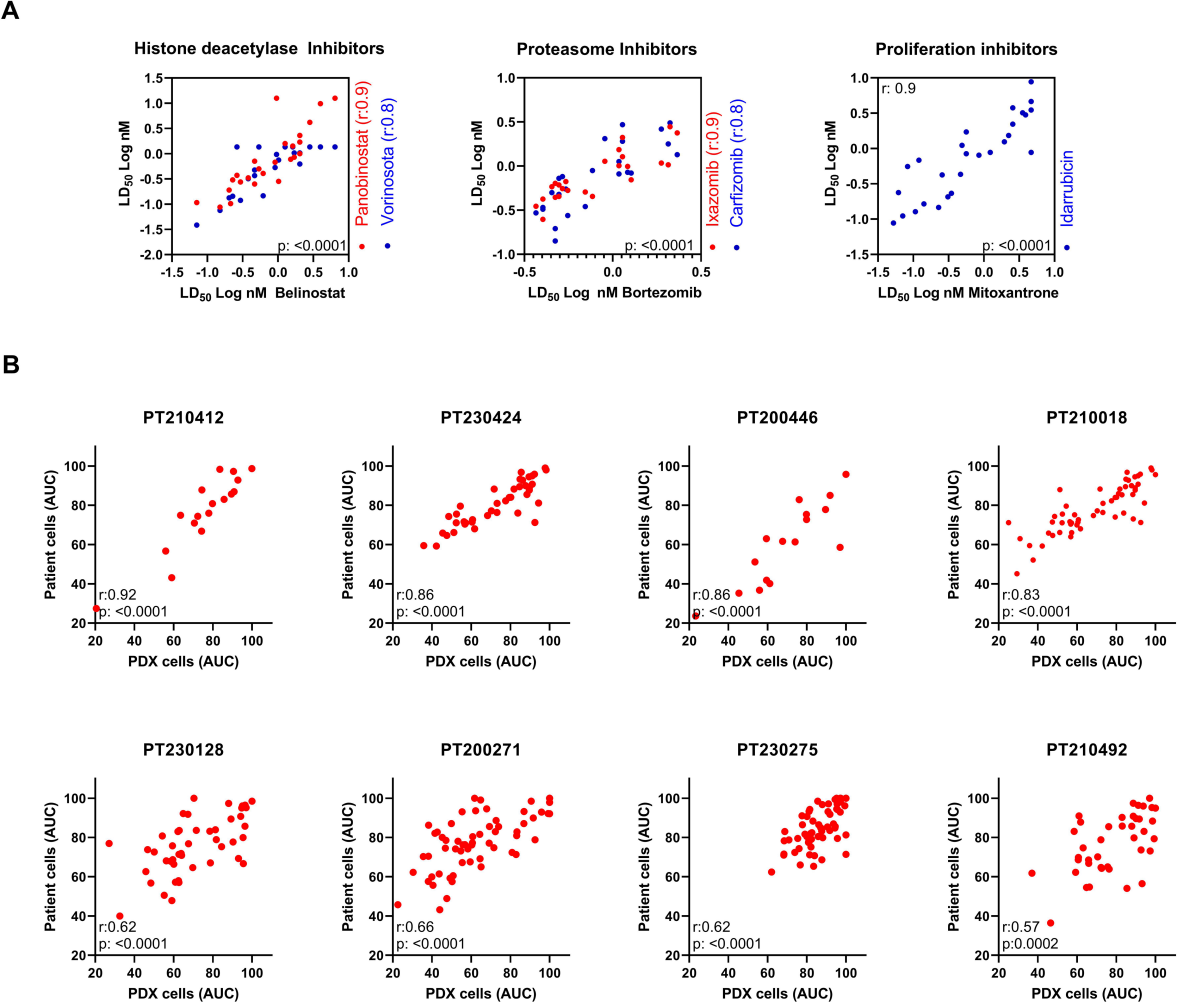
Correlation between drugs of the same class and between patient samples and their respective PDX samples. **(A)** Response to drugs of the same class. Correlation analysis of the LD_50_ for the 25 ALL samples against different drugs of the same class. Each circle corresponds to one ALL case. Correlation between the LD_50_ of different histone deacetylase inhibitor agents, proteasome inhibitor agents, and proliferation inhibitors. The Pearson’s correlation coefficient and *p*-value are shown. **(B)** Concordance of drug response profiles between isolated patient samples and matched PDX-derived leukemia cells. ELDA was performed on eight pediatric ALL samples and their matched PDX counterparts across a panel of 50 compounds. Drug response was quantified using the area under the dose-time–response curves (AUC). Each panel represents one patient–PDX pair, with each data point corresponding to the AUC value of a single drug measured in the patient and the matched PDX sample. Pearson correlation coefficients are indicated within the respective panels.

A positive correlation was observed between the response to drugs of the same class. For instance, histone deacetylase inhibitors such as belinostat, panobinostat, and vorinostat exhibited a positive correlation in drug response. Similarly, among the proteasome inhibitors, including bortezomib, ixazomib, and carfilzomib, a positive correlation in drug response was evident. Furthermore, a positive correlation was observed among the proliferation inhibitors, such as idarubicin and mitoxantrone (**Figure 6A**). These findings suggest a high level of accuracy of the method for predicting drug responses.

The use of PDX-derived leukemia samples may introduce potential biological biases, including clonal selection during engraftment, adaptation to the murine microenvironment, and partial loss of intrapatient heterogeneity. To directly address the concordance between fresh patient samples and their corresponding PDX, we compared drug response profiles from eight freshly isolated leukemia samples and their matched PDX counterparts across the 50-drug panel. While some quantitative differences in AUC magnitude were noted for individual compounds, a significant positive correlation was observed between AUC values obtained from fresh patient samples and their matched PDX-derived cells (**Figure 6B**). Thus, despite the potential biological shifts inherent to xenograft propagation, it does not fundamentally distort the drug response landscape captured by ELDA, as the overall drug response patterns were largely preserved. This finding supports the suitability of PDX-expanded material for standardized *ex vivo* drug profiling.

### 3.4 *In vivo* validation of ELDA

**Figure 7:**
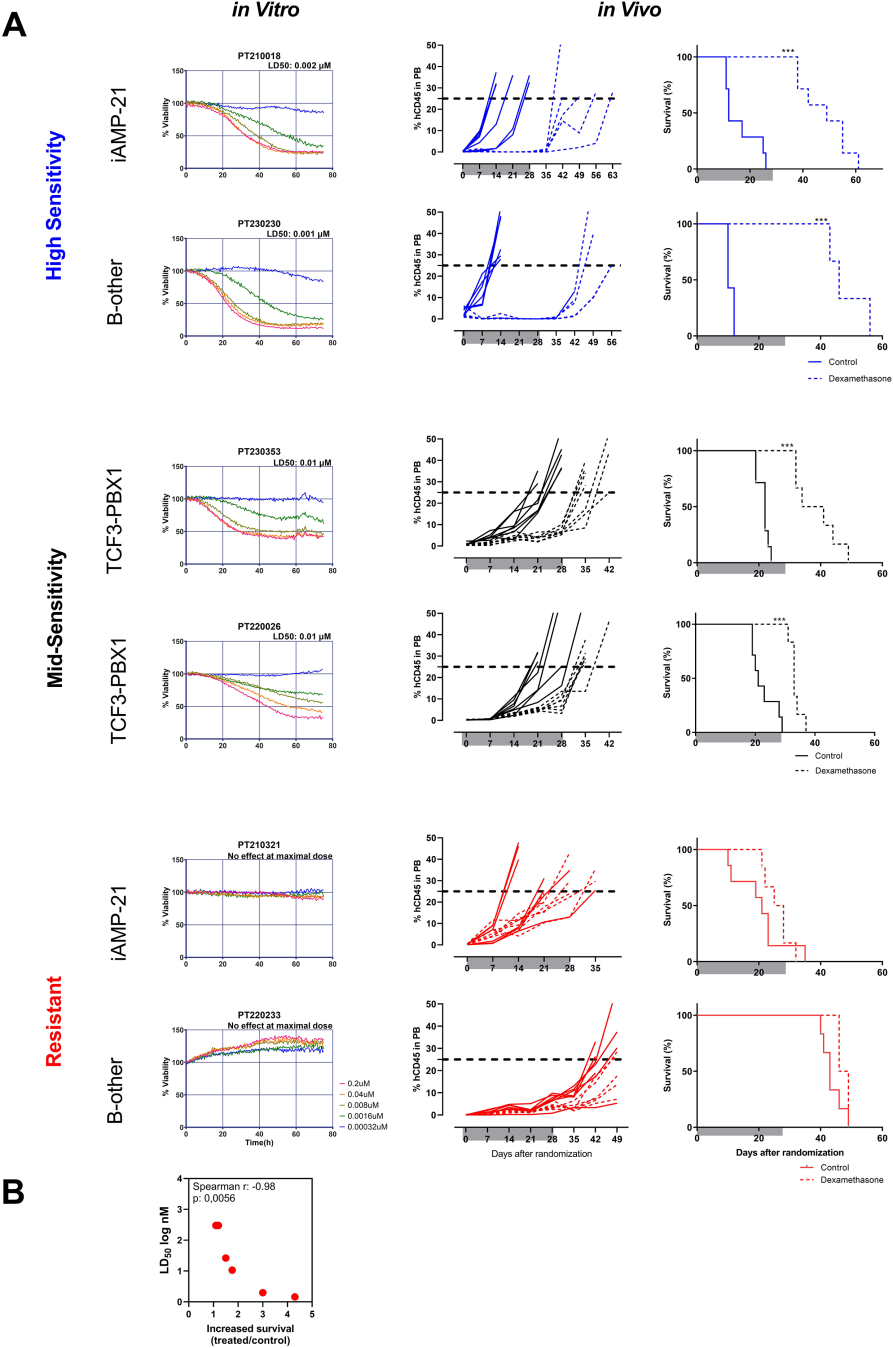
*In vivo* validation of ELDA results for dexamethasone. NSG mice were transplanted with three distinct pairs of ALL patient samples, each sharing the same genetic background (iAMP21, TCF3-PBX1, or B-other) but exhibiting different *in vitro* responses to dexamethasone. Animals were treated with dexamethasone (5 mg/kg intraperitoneally, Monday through Friday) for 4 weeks. **(A)** The left panels show ELDA-based *in vitro* sensitivity to dexamethasone for each patient, while the right panels present the corresponding *in vivo* data, including leukemia progression (percentage of hCD45^+^ cells in peripheral blood) and Kaplan–Meier survival curves. Shaded areas indicate the treatment period. Mice were considered to have reached an event when hCD45^+^ cells comprised 25% of peripheral blood leukocytes or when they exhibited leukemia-related morbidity (weight loss, lethargy, ruffled fur). Survival curves for the treated vs. control groups were compared using the log-rank test. **(B)** Correlation analysis was performed between the *in vitro* response to dexamethasone (LD_50_) and *in vivo* survival. If *in vitro* cell viability exceeded 50% at the highest drug concentration tested, the LD_50_ was set to twice the highest tested concentration. The leukemia growth delay ratio (*x*-axis) was calculated by dividing the median EFS of drug-treated mice by that of control mice.

To further validate the ELDA results, we analyzed *in vivo* responses from pairs of patients sharing the same genetic subtype but exhibiting different drug sensitivities (**Figure 7**). For example, xenograft PT210018 (i-AMP21) was highly sensitive to standard chemotherapy agents, including dexamethasone (LD_50_ = 0.2 nM) and vincristine (LD_50_ = 1.6 nM), whereas xenograft PT210321 (i-AMP21) was resistant to both drugs, showing no effect at the highest concentrations tested. Consistent with ELDA predictions, dexamethasone or vincristine treatment significantly prolonged the survival of mice transplanted with PT210018 leukemia (**Figure 7A****;** **Supplementary Figure 5**), while no survival benefit was observed in PT210321 (**Figure 7****;** **Supplementary Figure 5**). Clinically, patient PT210018 achieved a good response to induction therapy (MRD-negative at day 33), whereas patient PT210321 had a high MRD level (1.32 × 10**^−2^**) at the end of induction.

Similar concordance between ELDA and *in vivo* outcomes was observed in additional patient pairs: PT230353 *vs*. PT220026 (TCF3-PBX1) and PT230230 *vs*. PT220233 (B-other), for both dexamethasone (**Figure 7**) and vincristine (**Supplementary Figure 5**). For dexamethasone, the delay in leukemia progression *in vivo* showed a strong correlation with ELDA-derived LD_50_ values (Spearman ***r*** = −0.98, *p* = 0.0056; **Figure 7B**).

## 4 Discussion

Here, we present the standardization and validation of ELDA, a novel drug screening method based on multi-image bright-field microscopy. Parallel validation experiments of ELDA and other techniques, such as Calcein-AM and MTT assays, were performed. A strong correlation between the results was found, while several advantages of ELDA became evident. ELDA offers a significant reduction in the number of cells required for the assay, as it enables the screening of 31 drugs, at five concentrations, in duplicate, using only 1.5 million ALL cells. In ELDA, drug sensitivity is assessed under conditions that more closely resemble the physiological conditions, as ALL cells are cultured in the presence of stromal cells and in a collagen matrix that mimics the extracellular matrix in the bone marrow niche. ELDA is a non-invasive assay based on bright-field microscopy, requiring no cell viability marker substance, which may be toxic to cells or interfere with drug metabolism. Finally, unlike most cell viability assays, which are inherently destructive and limited to specific time points (25, 29, 37, 47, 48), ELDA monitors and measures the survival of cells continuously over time, providing a better evaluation of the kinetics of cell responses to drugs.

ELDA was also validated *in vivo* using two different PDX ALL cases of the same genetic background (i-AMP21) but with different responses to dexamethasone and vincristine. As expected, the higher the drug sensitivity in ELDA, the higher the survival of mice treated with the corresponding drug, supporting the translational relevance of the assay.

We tested 50 drugs on 25 ALL samples in the ELDA model. In general, compounds such as HDAC and proteasome inhibitors demonstrated efficacy at low LD_50_ values for most samples. Cytotoxic agents, such as dexamethasone, mitoxantrone, vincristine, dactinomycin, and venetoclax, displayed responses distributed over a wider concentration range, while most TKIs had no effect at the highest tested dose (5 μM) or demonstrated limited efficacy across ALL cases. These results seemed comparable to other drug screening studies in ALL (25, 29). One study, including 60 pediatric ALL samples, revealed that bortezomib exhibited effectiveness at low (IC_50_ < 10 nM) and narrow IC_50_ ranges, while venetoclax, dexamethasone, topotecan, and cytarabine displayed a wider concentration range, with varying degrees of activity observed in the nanomolar range across different samples. Additionally, some TKIs such as dasatinib and imatinib achieved either high or no IC_50_ values across most of the tested samples (25). Another study, with 805 pediatric ALL samples, demonstrated consistent efficacy of bortezomib and panobinostat in almost all samples within the nanomolar range. Conversely, compounds like venetoclax and cytarabine exhibited a more heterogeneous response, and dasatinib was either ineffective or displayed high IC_50_ values in the majority of samples tested, except for BCR-ABL1 and TCF3-PBX1 ALL cases (29).

Unfortunately, primary ALL cells tend to die when in culture, and this was not different in ELDA. In our experience, a quarter of ALL cases do not survive more than 48 h *in vitro*, and their drug response curves are not reliable. Preliminary experiment with different grow factors (IL3, IL7, SCF, FLT3L, IGFBP7, IGF1), adhesion molecules (CD40L, VCAM), SPITE Medium Supplement (insulin, transferrin, selenium, pyruvate, and ethanolamine, from Sigma-Aldrich S5666), and a lipid mixture 1 (non-animal derived fatty acids, cholesterol, Tween-80, tocopherol acetate, and Pluronic F-68, from Sigma-Aldrich L0288) render no difference, with the only exception of IL7 (data not shown). It is worth mentioning that stromal cells are able to move dead ALL cells, which may be a confounding effect, especially when ALL cells died too quickly. We recommend checking the time-lapse movies to identify this confounding effect. The ELDA software is under improvement to better discriminate cell death events using changes in cell attributes (49).

Recently, there has been a resurgence in drug profiling for primary ALL (25, 27, 29, 39, 50). Advances in PDX models of ALL and its validation provided sufficient number of ALL cells for the assays. *In vitro* drug testing using patient-derived or PDX cells has enabled the identification of novel therapeutic agents for ALL (25, 26). Large studies analyzing more than 600 ALL cases have demonstrated an association between *in vitro* drug-resistance profiles and clinical response (29, 51, 52), highlighting the clinical relevance of functional pharmacotypic profiling. Early approaches evaluated drug resistance in leukemic cells cultured in isolation, with viability determined using metabolic markers such as MTT, and these methods remain in use (29). More automated alternatives, such as fluorescein diacetate (53), and flow cytometry-based platforms enabling simultaneous phenotypic characterization in co-culture systems have further advanced the field (54–56). More recently, 3D co-culture systems incorporating mesenchymal and endothelial cells have been developed, revealing differences between 2D and 3D drug responses (25, 57, 58). However, these platforms do not provide real-time assessment of cell viability based on dynamic cellular behavior.

In this context, ELDA integrates stromal co-culture within a 3D matrix and defines viability through preserved cellular integrity (cellular refringence in bright-field microscopy) and sustained membrane motion over time. Because loss of membrane motion is an early event preceding apoptosis (33), ELDA may detect early apoptotic changes, although it may not fully distinguish intermediate states such as quiescence or sublethal drug effects. Our data further indicate that ELDA-derived drug–response profiles are preserved between freshly isolated patient samples and their matched PDX counterparts, despite potential biological shifts associated with xenograft propagation. ELDA therefore represents a promising and scalable functional drug profiling platform that may complement genomic classification and contribute to pharmacotyping strategies in pediatric ALL. To our knowledge, no independent studies evaluating ELDA have yet been published. The primary objective of this work was to establish and validate ELDA as a robust functional assay and translational research platform. Prospective trials integrating ELDA-guided interventions will be necessary to determine its clinical utility.

## Data Availability

The raw data supporting the conclusions of this article will be made available by the authors, without undue reservation.
