# Supplementary material for: ELDA: real-time functional drug profiling in acute lymphoblastic leukemia
Source: Front Oncol. 2026 Mar 20;16:1685447. doi: 10.3389/fonc.2026.1685447 (PMC13046488; doi:10.3389/fonc.2026.1685447)
Supplement: Supplementary file 1 [file DataSheet1.pdf]

frontiers

Supplementary Material

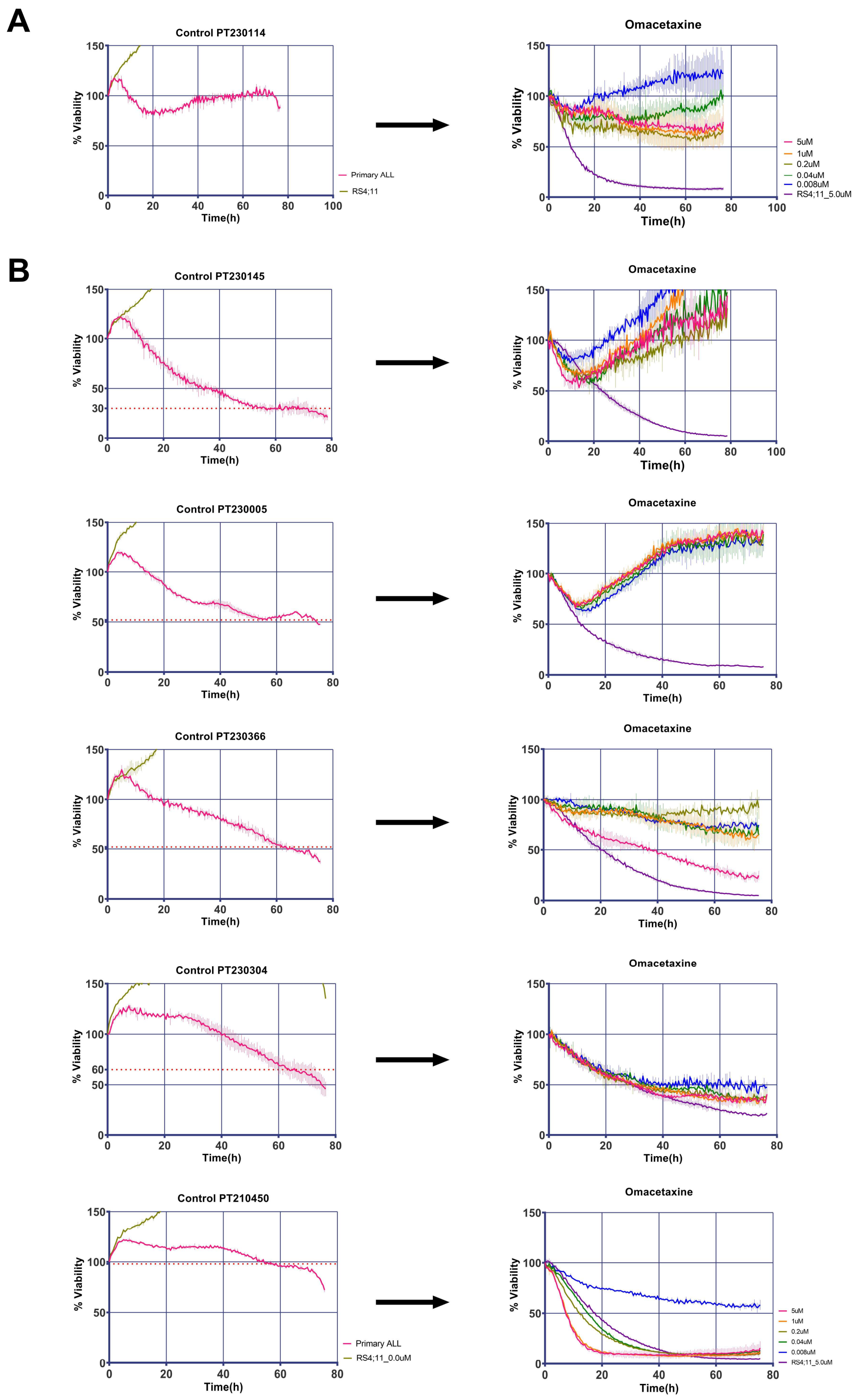

Supplementary Figure 1. ELDA quality control. (A) Example of a confounding scenario in which ALL cells initiated time-lapse acquisition largely nonviable (i.e., static). Note that both control viability appears acceptable (>60%) and the dose–response curves, although irregular, are not entirely erratic. However, these results are attributable to stromal cells passing near dead ALL cells and inducing their displacement, thereby artificially inflating viability estimates. Visual inspection of time-lapse movies is therefore mandatory to identify this artifact. (B) Representative examples illustrating the relationship between control viability and drug-response curves. PT230145 illustrates a clearly invalid experiment, with control viability at 60 h of approximately 30% and consequently inadequate drug-response curves. PT230005 shows control viability around 50% with erratic dose–response curves. PT230366, which also presents control viability near 50%, shows biologically interpretable drug-response curves, illustrating the need for case-by-case evaluation. PT230304 demonstrates control viability around 60% with preserved and coherent dose-dependent curves. Finally, PT210450 exhibits control viability >60% with robust drug-response behavior. Omacetaxine is shown as a representative compound to illustrate these patterns; however, similar curve patterns were observed across the majority of tested drugs. Together, these data support a three-step quality control process: (1) visual inspection of time-lapse videos, (2) analysis of the control viability curve, and (3) analysis of drug-response curves.

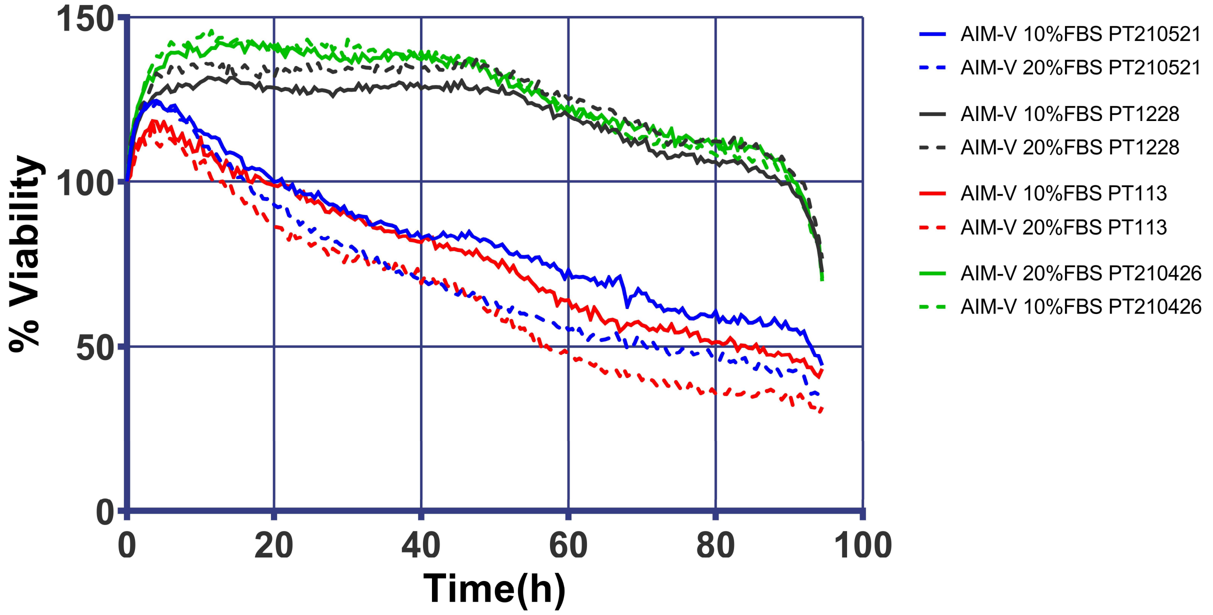

Supplementary Figure 2. Viability of ALL cells cultured in AIM-V 20% FBS versus AIM-V 10% FBS. Viability of four different ALL PDX cells after 96h of culture in AIM-V supplemented with 20% FBS versus AIM-V supplemented with 10% FBS. No major differences were observed, regardless of whether the samples maintained good or poor viability over time.

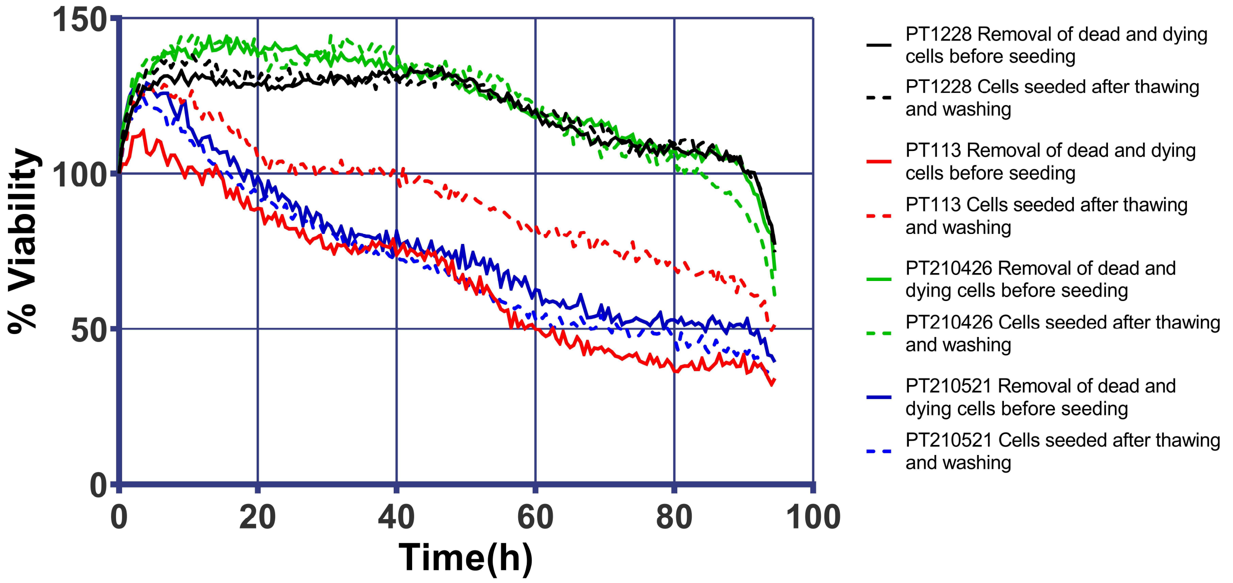

Supplementary Figure 3. Viability of ALL cells after the immunomagnetic removal of cell debris, dead cells, and dying cells. Four different patient PDX samples are shown. Dead and dying cells were removed using the Dead Cell Removal kit (Miltenyi). Removal of dead/dying cells before cell seeding made no difference in three samples, regardless of whether the samples maintained good or poor viability over time. Only in one sample removal of dead/dying cells prior to cell seeding, worsen viability.

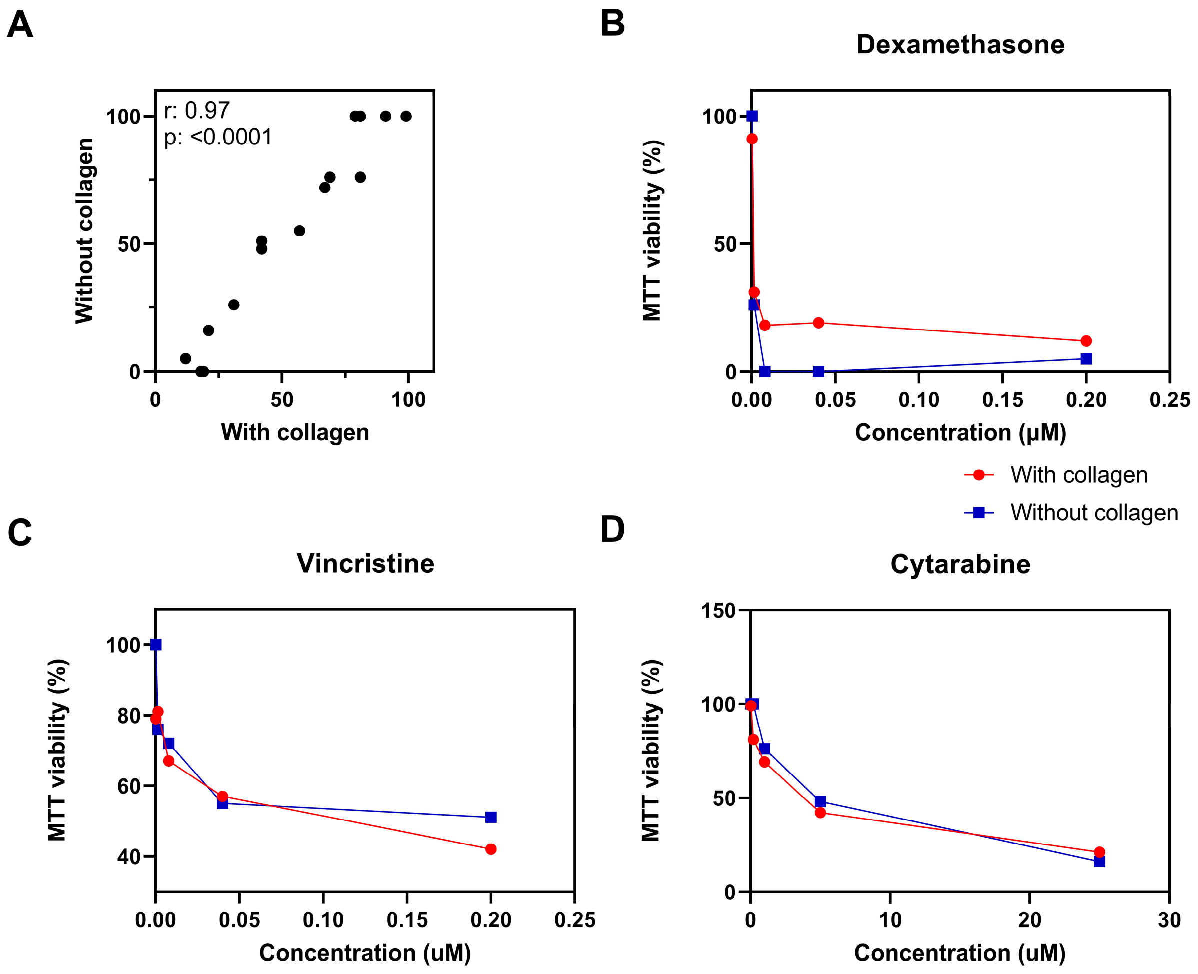

Supplementary Figure 4. MTT viability assay in the presence and absence of collagen. Results from a sample ALL PDX are shown. PDX cells were thawed and 100,000 cells were seeded per well in a 96-well plate, with or without collagen type 1 (1.5 mg/mL). After collagen polymerization (about 1h) 60 μL of RPMI1640 medium with 10% FBS and subsequently, drugs were added. After 72 h, the MTT reagent was added and formazan crystals were dissolved in acid SDS and the plate was read at 570 nm. (A) Correlation between MTT cell viability in the presence or absence of collagen. Each symbol corresponds to the mean of duplicate wells. Results are for cells treated with dexamethasone, vincristine, and cytarabine at five different concentrations or no drug. Pearson's correlation analysis is shown. Dose response curves for (B) dexamethasone, (C) vincristine, and (D) cytarabine, with and without collagen.

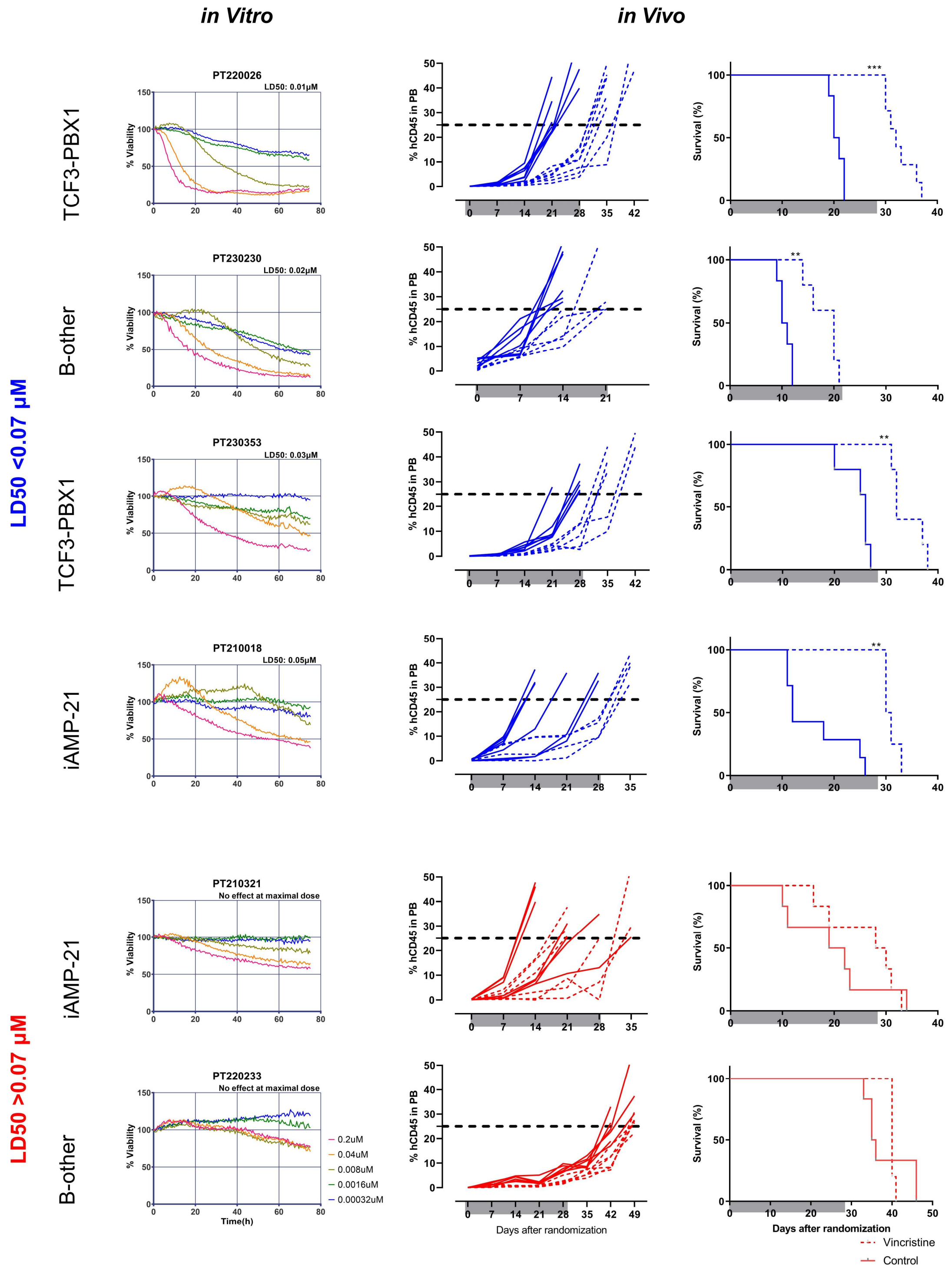

Supplementary Figure 5. In vivo validation of ELDA results for vincristine. NSG mice were transplanted with three distinct pairs of ALL patient samples, each sharing the same genetic background (iAMP21, TCF3-PBX1, or B-other) but exhibiting different in vitro responses to vincristine. Animals received vincristine (0.15 mg/kg, intraperitoneally, every Monday) for four weeks. The left panels show ELDA-based in vitro sensitivity to vincristine for each patient, while the right panels

present the corresponding in vivo data, including leukemia progression (percentage of hCD45+ cells in peripheral blood) and Kaplan–Meier survival curves. Shaded areas indicate the treatment period. Mice were considered to have reached an event when hCD45+ cells accounted for 25% of peripheral blood leukocytes or when they developed leukemia-related morbidity (weight loss, lethargy, ruffled fur). Survival curves for treated versus control groups were compared using the log-rank test. Samples are divided into two groups: the four upper panels show ALL samples with LD50 values below the median LD50 (0.07 □M) for vincristine among all samples analyzed in the study, while the lower two panels show vincristine-resistant cases, in which in vitro cell viability exceeded 50% at the highest drug concentration tested.

Supplementary Videos:

Supplementary Video 1. Example of an ELDA assay showing ALL cells remaining viable until the end of the experiment.

Supplementary Video 2. Example of an ELDA assay showing ALL cells undergoing a progressive loss of viability over the course of the experiment.

Supplementary Video 3. ELDA assay for idarubicin at 0.2 μM. Most drugs, including idarubicin, did not exhibit cytotoxic effects on T-MSCs at concentrations that were lethal to ALL cells.

Supplementary Video 4. ELDA assay for bortezomib at 0.2 μM. Bortezomib, carfilzomib, fedratinib, ixabepilone, omacetaxine, panobinostat, and vincristine showed cytotoxic effects on T-MSCs at the highest concentration tested.
